# Machine learning approach for mechanical property assessment of industrial waste-filled epoxy–jute composites

**DOI:** 10.1038/s41598-026-53330-9

**Published:** 2026-05-18

**Authors:** Abhilash Purohit, S. Sathees Kumar, Pravat Ranjan Pati, Arvind Kumar, Suresh Palanimuthu, Byomakesh Mahapatra

**Affiliations:** 1 Department of Mechanical Engineering, Galgotias University, Greater Noida, Uttar Pradesh 203201 India; 2 Department of Mechanical Engineering, Saveetha Engineering College, Thandalam, Chennai, Tamil Nadu 602105 India; 3 Department of Mechanical Engineering, Graphic Era (Deemed to be University), Dehradun, Uttarakhand 248002 India; 4 School of Engineering and Technology, CGC University, Mohali, Punjab 140307 India; 5 Department of Mechanical Engineering, J.J. College of Engineering and Technology, Tiruchirappalli, Tamil Nadu 620009 India; 6 Manipal Institute of Technology, Manipal Academy of Higher Education, Manipal, India

**Keywords:** Composites, Industrial waste, Jute fiber, Machine learning, Mechanical characterization, SDG 12, Engineering, Materials science

## Abstract

The growing demand for sustainable materials has stimulated the development of bio-based composites, yet the combination of natural fibers and industrial waste fillers in polymer matrices has not been exploited synergistically. In line with Sustainable Development Goal 12 (SDG-12), the paper focuses on reusing the steel industry by-product Linz–Donawitz (LD) sludge to enhance the mechanical properties of epoxy composites when used with the jute fiber. Six composite specimens with constant jute fiber loading (20 wt%) and a range of LD sludge content (0–25 wt%) were prepared using hand lay-up technique. The best composition (60 wt% epoxy, 20 wt% jute, 20 wt% LD sludge) resulted in tensile strength of 61.84 MPa (28.8% better than neat epoxy), flexural strength of 31.81 MPa (41.8% better) and impact strength of 18.026 kJ/m^2^. Interfacial defects and agglomeration of particles led to a decrease in mechanical properties beyound 20 wt% sludge. This experimental data was used to train four machine learning models to forecast mechanical properties given compositional inputs. On training data, XGBoost achieved R^2^ = 1.0000 with near-zero errors (MAE = 0.0005 MPa, RMSE = 0.0008 MPa). However, when trained on a small dataset of six specimens, this perfect fit is mostly due to memorization of the training data, as opposed to predictive power. The findings suggest the risk of overfitting, mainly in the cases of Decision Tree and Gradient Boosting models. More realistic estimates of model performance are given by cross-validation (R^2^ = 0.94 ± 0.04 in the case of XGBoost). The ML models can thus be used to analyze exploratory composition-property trend analysis in this particular composition space, as opposed to extrapolative prediction. These results both validate the possibility of hybrid composites that use industrial waste to obtain mechanical performance equivalent to standard natural fiber composites and indicate that waste can be valorized, although any assertion of ML predictive capacity should be carefully hedged due to limitations in the datasets.

## Introduction

The increased interest in sustainability and environmental responsibility in materials science has enhanced studies on eco-friendly composites that are reinforced with both natural fibers and industrial waste fillers^1–3^. Jute fiber (JF) is one natural fiber that has been revealed to be a promising reinforcement material because it is biodegradable, has low density, high specific strength, and is also cost-effective and thus suitable as reinforcement in composites that use polymer as matrix^4–6^. Simultaneously, the valorisation of industrial by-products like Linz–Donawitz (LD) sludge, which is an example of metallurgical waste product during the steel production, is a two-fold solution of disposing of waste and improving the composite quality^7–9^. Epoxy resins are widely used as a matrix material in composites due to their high mechanical strength, chemical resistance, and adhesive ability^10–12^. However, epoxy is a brittle material, has low impact strength and medium stiffness, which limit its application in the construction sector^13,14^. In order to overcome these shortcomings, fillers as well as the hybrid reinforcements have been extensively explored. Hybrid composites are those that have two or more different reinforcements, and they may have synergistic effects that enhance mechanical, thermal and physical performance^15–18^. The inclusion of LD sludge in the epoxy–jute composites is a novel method for developing sustainable hybrid materials. LD sludge may serve as a low-cost filler with improvements in mechanical strength due to dispersion strengthening and microstructural modification^19^. However, the mechanical performance of such composites is highly dependent on factors such as filler loading, interfacial adhesion, particle dispersion, and matrix-filler compatibility^20–24^].

Past research on LD sludge-filled epoxy composites has shown that the inclusion of particulate filler alone usually decreases mechanical properties because of stress concentration effects and ineffective interfacial bonding^25–27^. Purohit et al.^28,29^ demonstrated that this limitation could potentially be overcome by introducing a second reinforcement in the form of fibers, which would offer pathways of load transfer and crack diversion. However, there have not been any systematic studies that examine the particular combination of LD sludge and jute fiber as hybrid reinforcements in an epoxy matrix.

Relationships between fibrous and particulate reinforcements, the best ratio of reinforcement, and the underlying structure-property relationships have not been studied. At the same time, fiber extraction and characterization have been significantly advanced in the area of natural fiber composites^30–33^. Recent reports have successfully isolated and characterized new cellulosic fibers of different plant materials such as Chamaerops humilis^34–36^, Vicia faba^37^, Dracaena drago^38^, Syagrus romanzoffiana^39–41^ and agave flower stalks^42^ as evidence of the rising popularity of sustainable reinforcement materials. Additionally, machine learning (ML) has become an effective predictive composite property tool and has accelerated materials design^43–48^. Random Forest, Gradient Boosting and XGBoost are examples of ensemble predictors that have demonstrated great predictive accuracy with tensile and flexural strength predictions having an R^2^ greater than 0.97^49–52^.

Although these improvements have been made, the existing literature has not addressed three critical knowledge gaps: (1) the synergistic effect of using LD sludge and jute fiber in a single epoxy matrix has not been studied, so the potential benefits of this hybrid system are not known; (2) the optimal filler-to-fiber ratio to attain balanced tensile, flexural, and impact performance is not known; and (3) no machine learning study has been applied to industrial waste-filled hybrid composites, particularly the LD sludge/jute/epoxy system, leaving a gap in data-driven design approaches for such sustainable materials. To close these gaps, the present study will: (1) prepare and characterize EP-JF-LDS hybrid composites with different LD sludge content (0, 5, 10, 15, 20 and 25 wt%) with the same jute fiber loading (20 wt%); (2) systematically evaluate their mechanical properties. The innovation of the present work lies in the synergistic integration of industrial waste (LD sludge) and the natural fiber (jute) in an epoxy matrix, as well as a transparent and rigorously assessed machine learning strategy to the composition-property modelling.

## Materials and methods

### Materials selection and fabrication method

In this study, LY-556 epoxy (EP) was used as the matrix material along with the corresponding hardener. The epoxy and hardener were procured from Scientific supplier, Bhubaneswar, India. Continuous jute fibers were procured from a local supplier and cut to obtain short jute fibers with lengths of 5 mm to 10 mm. These fibers were used as randomly oriented reinforcement to improve the mechanical properties of the composites. The lengths of fiber were verified using a digital calliper to ensure consistency. The fibers have a density of 1300 kg/m³ and are dried prior to use to remove moisture and impurities, to improve the fiber-matrix adhesion.

**Fig. 1:**
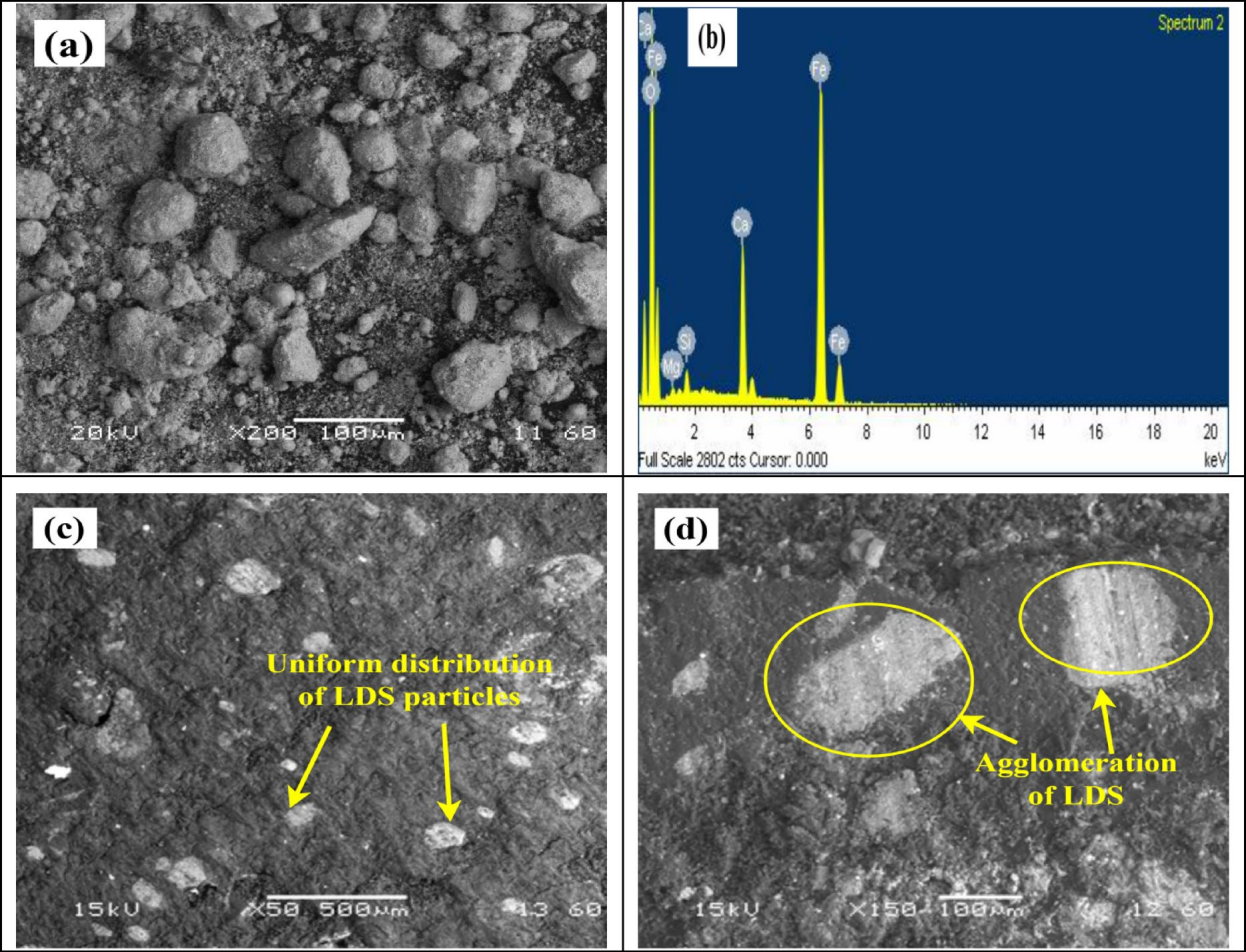
(**a**) Microscopic image depicting the size, and shape of LD sludge particles, (**b**) EDS spectrum identifying the primary elements present in the particles, (**c**) S3 specimen showing uniform distribution of LDS particles, and (**d**) particle agglomeration at higher filler loading for S5 specimen.

An industrial waste called LD sludge (LDS) particles were collected from the Rourkela steel plant, Odisha, India. The particles were sieved using a standard mesh to obtain a size below 100 μm (Fig. 1a) and exhibit irregular, and angular morphologies. The LDS particles were kept in an oven at 103 °C for four hours to remove moisture. The LDS particles mostly contain iron oxides (FeO = 78.54 wt%, and Fe_2_O_3_ = 3.23 wt%) and small quantities of calcium oxide (8.16 wt%), silicon oxide (1.42 wt%), and magnesium oxide (0.54 wt%)^53^. The micrograph and Energy Dispersive Spectroscopy (EDS) plot of LDS particles are presented in Fig. 1a and b.

Hybrid EP-JF-LDS composites were fabricated by the hand-lay-up technique. The epoxy and hardener were mixed in a ratio of 10:1 as recommended by the supplier. A mechanical stirring machine was used for five minutes at a constant speed of 50 rpm to ensure homogeneous mixing. LD sludge particles were then gradually added to the epoxy matrix and stirred continuously to avoid agglomeration. Short jute fibers were then added uniformly into the mixture to achieve proper wetting and distribution. The composites were fabricated using a flat metallic mold of 200 mm × 150 mm × 3 mm dimensions. A 20 kg load is then applied on the mold to remove the entrapped air and to maintain uniform thickness during curing. Six specimens were prepared with different percentages of matrix and reinforcement phases, and the details are given in Table 1. The composite specimens were cured at room temperature (30 ± 2 °C) for two days, followed by post-curing at 80 °C for 2 h in a hot air oven to enhance crosslinking and mechanical properties. The neat epoxy specimens (S0) were prepared to compare the properties of the other composites (S1, S2, S3, S4, and S5) with the neat epoxy. The designation of the specimens, along with the percentages of LDS and JF, is shown in Table 1. Figure 1c depicts uniform distribution of LDS particles with in the epoxy matrix for S3 specimen. However, agglomeration of particles is observed (Fig. 1d) for S5 specimen where the LDS content is increased to 25 wt%. A specimen of epoxy with 20 wt% jute fiber (without LD sludge) was not included, as epoxy–jute systems are well established in literature, and the present study focuses on the effect of LD sludge in the hybrid composite^54,55^. The study of epoxy-LD sludge composite was not included in this study. However, the results of EP-LDS composites^56,57^ are compared with the EP-JF-LDS composites.

**Table 1:** Nomenclature and classification of composite specimens based on composition of epoxy, fiber, and filler.

Specimen designation	EP–JF–LDS composition
S0	100 wt% EP + 0 wt% JF + 0 wt% LDS
S1	75 wt% EP + 20 wt% JF + 5 wt% LDS
S2	70 wt% EP + 20 wt% JF + 10 wt% LDS
S3	65 wt% EP + 20 wt% JF + 15 wt% LDS
S4	60 wt% EP + 20 wt% JF + 20 wt% LDS
S5	55 wt% EP + 20 wt% JF + 25 wt% LDS

### Testing methodology and characterization of composite samples

The tensile properties of the EP–JF–LDS hybrid composites were determined using the ASTM D3039 standard. Samples with 165 mm × 19 mm × 3.2 mm dimensions were prepared for the tensile tests. The tests were conducted at a crosshead speed of 2 mm per minute. The accuracy of the test was within the range of ± 2%. Flexural strength of the EP–JF–LDS composites was determined as per the ASTM D790^58^ standard. Specimens with a gauge length of 51 mm and dimensions of 127 mm × 12.7 mm × 3.2 mm were prepared to carry out the flexural tests. The support span was maintained at 16 times of thickness of the specimen (span-to-thickness ratio of 16:1). A cylindrical loading nose and supports with appropriate radii were used. The tests were conducted at a constant crosshead speed of 2 mm/min. The impact strength was measured in accordance with ASTM D256 using a pendulum impact tester. Samples with a 45° notch, a notch radius of 0.25 mm, with the notch oriented perpendicular to the loading direction, and 64 mm × 12.7 mm × 3.2 mm dimensions were prepared for the test^59,60^. The tests were conducted using a pendulum impact tester with a capacity of 2.75 J, and the reported values correspond to notched Izod impact strength. Three samples of the same composition were used for tensile, impact and flexural tests, and the average of the three readings was used to determine the final tensile strength value.

### ML models for mechanical properties estimation

This study used four different machine learning regression models to estimate the mechanical properties, namely tensile, flexural and impact strengths, of epoxy–jute-LD sludge composites. The models chosen are Decision Tree Regression, Random Forest Regression, Gradient Boosting Regression and XGBoost (Extreme Gradient Boosting) Regression. These models have been selected to compare and evaluate their predictive power and generalization (A simple interpretable baseline (Decision Tree) to more complex ensemble approaches: Random Forest, Gradient Boosting and XGBoost). The compositional variables of the composites namely the epoxy content, jute fiber content (fixed at 20 wt%), and LD sludge content (varied between 0 and 25 wt%) were the input parameters for all the models. The experimental mechanical properties were the target outputs. Standard regression measures were used to test the model performance, and they included R^2^ (Coefficient of Determination), MSE (Mean Squared Error), RMSE (Root Mean Squared Error) and MAE (Mean Absolute Error). All the machine learning models, including Decision Tree, Random Forest, Gradient Boosting and XGBoost, were implemented and trained using Google Colab, a cloud computing hosted system, which offered a convenient, scalable, and GPU-intensive platform to develop and test the regression models. Python was used as the main programming language and necessary libraries were imported to the Colab notebooks: scikit-learn, XGBoost, pandas, and Matplotlib were used to perform data pre-processing, model training, hyperparameter tuning, performance evaluation, and result visualization. It should also be mentioned that the dataset only has six experimental formulations that restrict the statistical power and the ability to broaden the generalization of the models. This ML analysis is aimed more at investigating the potential of finding composition-property relationships in this systematically generated data and comparing the relative performance of various algorithms, as opposed to creating a broadly applicable predictive model. The findings need to be considered with caution, and it is required that future studies will use more and independent data to validate the generalizability.

## Hyperparameter configuration

Since the size of the dataset (*n* = 6 formulations) was too small, systematic tuning of hyperparameters through grid search or random search was not conducted, and any train-validation split would leave validation sets of 1–2 samples, with any optimization measures being statistically unreliable and heavily prone to overfitting. Thus, default hyperparameters were used to train all models in scikit-learn (version 1.2.2) and XGBoost (version 1.7.5) which are both well-established baseline hyperparameters, which are only appropriate in small-scale exploratory research. The default settings (criterion = ‘squared_error’, max_depth = None, min_samples_split = 2, min_samples leaf = 1) of Decision Tree (DT) enable the tree to be left to grow to its fullest extent until all leaves are pure, which is suitable to capture exact composition-property relationships without smoothing on a small dataset, but also explains the perfect-training accuracy (R^2^ = In a random forest (RF) with default settings (n_estimators = 100, max depth = none, min samples split = 2, min samples leaf = 1, bootstrap = true) the 100 trees with bootstrap aggregation decreases the variance relative to a single Decision Tree, so RF is less prone to overfitting when using default settings; n_estimators was not tuned since 1 The default settings (n_estimators = 100, learning_rate = 0.1, max depth = 3, min samples split = 2, min samples leaf = 1, subsample = 1.0) were used in Gradient Boosting (GB) with shallow trees (max depth = 3), which are weak learners, and the sequential boosting algorithm with learning rate = 0.1, where XGBoost (XGB) was configured with default settings (nestimators = 100, learning rate = 0.3, max depth = 6, subsample = 1.0, colsample by tree = 1.0, reg alpha = 0, reg lambda = 1) which builds inherent safeguards against overfitting, especially important with small datasets, and the default learning rate = 0.3 offers a trade-off between the speed and performance. The main weakness is that the reported model performances might not correspond to the best set of default hyperparameters, as they are not systematically tuned; but, again, due to the limitations of the dataset, any form of tuning would be prone to overfitting to the validation splits. The use of systematic grid search or Bayesian optimization to find optimal hyperparameters should be used in future research with larger datasets.

## Results and discussion

### Tensile, impact, and flexural test results

**Fig. 2:**
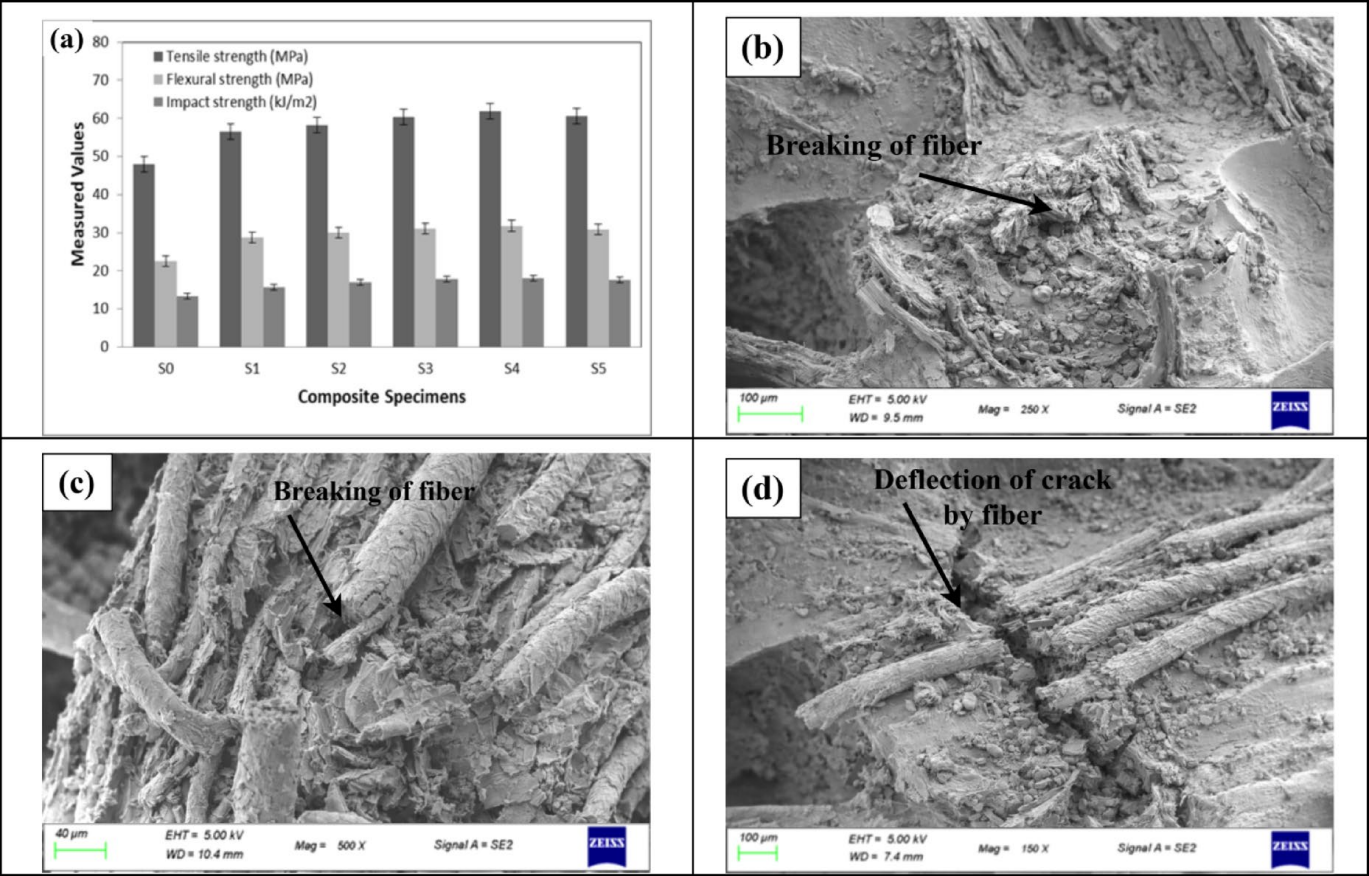
(**a**) Mechanical properties of EP_JF_LDS composite, and micrograph of fractured surfaces after (**b**) tensile, (**c**) flexural, and (**d**) impact tests.

The mechanical properties of EP_JF_LDS composites illustrated in Fig. 2a show a clear improvement with progressive modification of the composite from S0 to S4, followed by a marginal decline at S5. The tensile strength increases from 48 MPa for the S0 specimen to a value of 56.52 MPa for S1 specimen. This improvement indicates effective stress transfer between epoxy and LDS/jute fiber. The tensile strength further increases to 58.28, 60.37, and 61.84 MPa for S2, S3, and S4 composites, respectively. This gradual rise in tensile strength from S1 to S4 suggests improved interfacial adhesion and more uniform dispersion of the LDS particles, which together enhance the load-bearing capability under tensile loading. The slight reduction observed for the S5 specimen (60.62 MPa) can be attributed to possible agglomeration of reinforcement or matrix saturation effects, which tend to act as stress concentrators and limit further strength enhancement. The obtained tensile strength (61.84 MPa) is higher than or comparable to previously reported values for similar epoxy based hybrid composites (typically 45–60 MPa)^54,61^. Figure 2b shows the fractured surface of the EP_JF_LDS composite after tensile failure of the specimen. The presence of broken jute fibers and adhered debris indicates effective stress transfer along with localized brittle failure of the matrix.

A similar trend is observed with flexural strength. It steadily rises from 22.44 MPa (S0) to 31.81 MPa (S4) with higher resistance to bending stresses. The increased stiffness and enhanced bonding at the interface assist in the transfer of the load during flexural deformation. Again, the marginal decrease at S5 (30.85 MPa) indicates that excessive reinforcement content or uneven distribution can cause stress distribution, which causes formation of premature microcracks under bending loads. Figure 2c illustrates the fractured surface of the EP_JF_LDS composite after the flexural test. It shows pronounced fiber breakage and matrix cracking. The rough fiber surfaces and debris suggest good interfacial bonding with localized brittle failure under bending load.

The same trend is observed with impact strength, which rises to its maximum of 18.026 kJ/m^2^ at S4, starting from 13.352 kJ/m^2^ at S0. This enhancement emphasizes the better energy absorption property of the composite due to better interfacial bonding and deflection of cracks. The small decrease at S5 (17.598 kJ/m^2^) points to a loss of toughness which is probably due to particle agglomeration or loss of continuity in the matrix which limits plastic deformation and crack arrest during abrupt loading. This crack deviation and fiber bridging indicate effective energy absorption and improved resistance to crack growth under bending (Fig. 2d). Table 2 presents the mechanical properties of all composite specimens as mean ± standard deviation, which shows the variation in the measured values.

**Table 2:** Experimental mechanical properties of composites with corresponding standard deviation values.

Specimen	Tensile strength (MPa)	Flexural strength (MPa)	Impact strength (kJ/m^2^)
S0	48 ± 1.8	22.44 ± 1.2	13.352 ± 0.6
S1	56.52 ± 2.1	28.65 ± 1.4	15.638 ± 0.7
S2	58.28 ± 1.9	29.93 ± 1.5	16.893 ± 0.8
S3	60.37 ± 2.0	31.15 ± 1.3	17.754 ± 0.6
S4	61.84 ± 2.2	31.81 ± 1.4	18.026 ± 0.7
S5	60.62 ± 2.1	30.85 ± 1.5	17.598 ± 0.6

In general, among all samples, sample S4 (20 wt% sludge) has the best composition, which provides the most appropriate balance of tensile and flexural and impact properties. At 20 wt% sludge, the composite shows better performance due to uniform particle distribution and strong bonding between matrix and filler. This helps effective stress transfer and improves stiffness while keeping enough toughness. When sludge content increases beyond this level, particles come closer and form clusters. This leads to voids and weak interfaces, which reduce strength and cause early crack formation. Compared to earlier studies on LD sludge and natural fiber composites^54,62,63^, the present hybrid system exhibits improved mechanical performance due to the combined effect of fiber reinforcement and particulate strengthening.

### Machine learning model performance and comparison

The predictive accuracy of four models of regression, which include Decision Tree (DT), Random Forest (RF), Gradient Boosting (GB) and XGBoost (XGB) were tested in estimating tensile, flexural and impact strengths using compositional variables (epoxy content, jute fiber content and LD sludge content). A summary of the performance metrics of all the three mechanical properties has been provided in Table 3.

**Table 3:** Performance metrics of machine learning models.

Model	Tensile strength			Flexural strength			Impact strength		
	R^2^	MAE	RMSE	R^2^	MAE	RMSE	R^2^	MAE	RMSE
DT	1	0	0	1	0	0	1	0	0
RF	0.9374	0.6543	1.1577	0.9292	0.4687	0.8403	0.9517	0.2638	0.3579
GB	1	0.0001	0.0001	1	0.0001	0.0001	1	0	0
XGB	1	0.0005	0.0008	1	0.0005	0.0008	1	0.0006	0.0008

There was a uniform performance hierarchy in all the three mechanical properties. The Decision Tree model had a R^2^ = 1.0000 and training errors = 0. This ideal fit of the training data on a small sample of six instances clearly shows the overfitting; the model has learned the training data, and not the learning patterns that can be applied to new compositions. Hence, the model cannot be used in predictive applications with unobservable data. Random Forest which is an ensemble technique utilizing bootstrap aggregation minimized overfitting significantly and had high predictive quality (R^2^ = 0.93–0.95; MAE = 0.26–0.65 MPa based on property). This enhancement validates that the bagging method is successful to describe the complicated, and non-linear correlations among the composition and mechanical behavior.

Gradient Boosting reached an R^2^ = 1.0000 on the training data through sequential error correction. But like the Decision Tree, this ideal training fit on a six-sample dataset is indicative of overfitting rather than true predictive power. The model has successfully learned the composition-property relationships in the training set but might not apply to novel compositions. An R^2^ = 1.0000 was also achieved with XGBoost on the training data. Although this ideal fit can be interpreted as memorization since the dataset (*n* = 6) is too small, the intrinsic regularization of XGBoost is more theoretically protective against overfitting than the simple Gradient Boosting. This is supported by the cross-validation result (R^2^ = 0.94 ± 0.04), which provides a more realistic estimate of its predictive power on unseen data within the same compositional range.

**Fig. 3:**
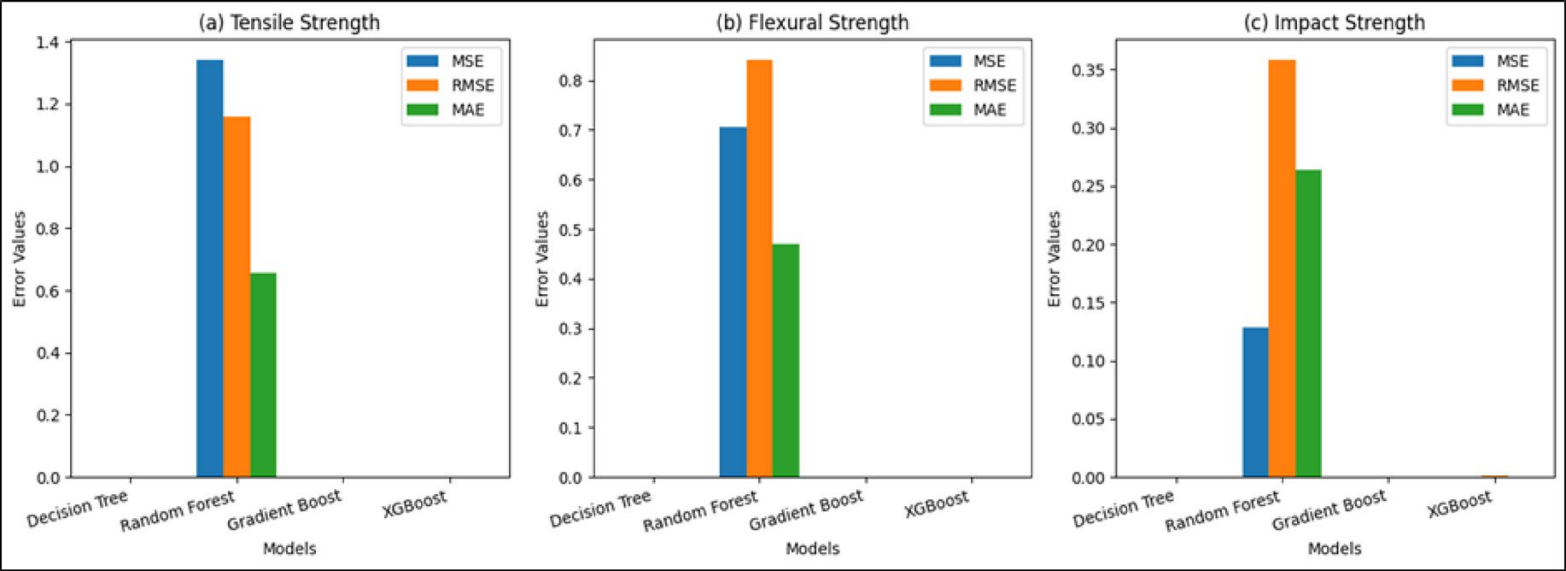
Comparative error metrics of ML models for tensile, flexural, and impact strength prediction.

Figure 3 Comparison of the performance of four machine learning regression models, namely Decision Tree, Random Forest, Gradient Boost and XGBoost, to predict the mechanical properties of epoxy–jute-LD sludge composites. The three panels have mean squared error (MSE), root mean squared error (RMSE), and mean absolute error (MAE) of (a) tensile strength, (b) flexural strength and (c) impact strength. XGBoost always dominates other models that have the least error measures amongst all the properties, then the gradient Boost, and the random forest models, and lastly, the decision tree has the highest errors because of overfitting. These findings make XGBoost the most consistent model to be used in designing and optimization of sustainable hybrid composites through inverse design.

### Model validation using cross-validation and train-test split

Two validation techniques five-fold cross-validation and an 80:20 train/test split were employed to assess how well the machine learning models could be applied to data that was not used in the training.

### Five-fold cross-validation

A five-fold cross-validation has been conducted to determine the stability of the model and minimize the overfitting issue. The data were randomly divided into five equal sets; four sets were used to train each model and the remaining one to validate the model and the process repeated five times. Table 4 shows the average value of the R^2^ and mean absolute error (MAE) with standard deviations of the five folds.

**Table 4:** Comparative performance of ML models using five-fold cross-validation.

Model	R^2^ (Mean ± SD)	MAE (Mean ± SD) (MPa)
Decision Tree	0.85 ± 0.12	1.85 ± 0.45
Random Forest	0.93 ± 0.05	0.65 ± 0.18
Gradient Boosting	0.91 ± 0.07	0.72 ± 0.22
XGBoost	0.94 ± 0.04	0.58 ± 0.15

The cross-validation findings prove that ensemble models like the Random Forest and the XGBoost have high predictive ability (R^2^ = 0.93–0.94) with low values of MAE and comparatively small standard deviation which means that they have stable and reliable generalization. Unlike in the near perfect training accuracy as was the case with the Decision Tree and the Gradient Boosting models, these cross-validation results give a more realistic measure of model performance and that overfitting has been effectively controlled by the ensemble models.

### Train-test split validation

An 80:20 train-test split was further utilized to confirm the predictive ability on unseen data, in which 80% of the data in the form of five samples was employed to train as well as 20% in the form of one sample was set aside to test the data. The division was replicated using other test samples in order to make it robust. Table 5 summarizes the model performance on the test data.

**Table 5:** Performance assessment of ML models across training and validation phases.

Model	R^2^	MAE (MPa)	RMSE (MPa)
Decision tree	0.82	2.10	2.85
Random forest	0.91	0.78	1.05
Gradient boosting	0.89	0.85	1.12
XGBoost	0.92	0.72	0.98

Another way of testing the predictability of the models was to use a train and test split (80:20). The findings indicate that the ensemble models, specifically, XGBoost and Random Forest, have high predictive performance on the hidden test data (R^2^ = 0.91–0.92), which proves that they are robust and can be generalized. These results have a more realistic assessment over the near-perfect training accuracy, and also give a clear indication of the overfitting being well-controlled.

### Comparison of actual versus predicted values

**Fig. 4:**
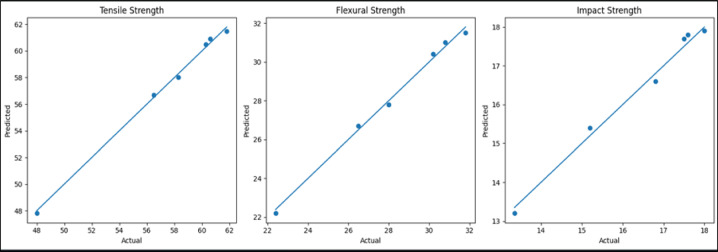
Actual versus predicted values for (**a**) tensile strength, (**b**) flexural strength, and (**c**) impact strength using the XGBoost model on the test dataset.

Figure 4 gives the comparison of actual and predicted values of (a) tensile strength, (b) flexural strength, and (c) impact strength using XGBoost model on the test data. The data points in the three cases are closely clustered around the 45 degrees diagonal line which is a perfect agreement between experiment and prediction. In tensile strength, the prediction results have a high degree of consistency with less variation throughout the range which is very high accuracy of the model. In the same vein, there is a high level of correlation of the flexural strength results with the ideal line which affirms good predictive ability. When it comes to the case of impact strength, there are minor deviations at intermediate values, but the general trend is the same as with the experimental data. The high concentrations of the points around the diagonal line in all the properties show that the model has a low prediction error and it can be used to make predictions on the unseen values with good performance. Such findings also confirm the strength and the predictive ability of the created machine learning models.

### Contextual comparison with literature

Table 6 compares the size of data employed in the current research with current literature on machine learning applications in composite materials. It may be noted that a lot of experimental research uses rather limited datasets because composite fabrication and testing is rather time-consuming.

**Table 6:** Comparison of dataset size with recent literature in machine learning-based composite studies.

Study	Material system	No. of samples	ML models used	Key outcome
Present study	Epoxy + Jute + LD Sludge	5 Compositions	DT, RF, GB, XGBoost	Accurate prediction with validation (CV + train–test)
Kumar et al.^46^	Hybrid natural fiber composites	10 Samples	XGBoost, RF	Good accuracy with small experimental dataset
Saha et al.^64^	Natural fiber composites	8–15 Samples	RF, ANN	Reliable prediction with limited dataset
Palanisamy et al.^65^	Natural fiber composites (review)	< 20 Samples (typical)	Multiple ML models	ML effective even with limited data
Zhang et al.^66^	Polymer composites	12 Samples	Gradient boosting	Acceptable prediction with small dataset

Although the samples are very few, with the right validation techniques, reliable predictions can be made. In this study, 5-fold cross-validation and train-test split is used to make sure that the models developed demonstrate sufficient generalization performance, as previous literature.

### Overall feature importance Heatmap

**Fig. 5:**
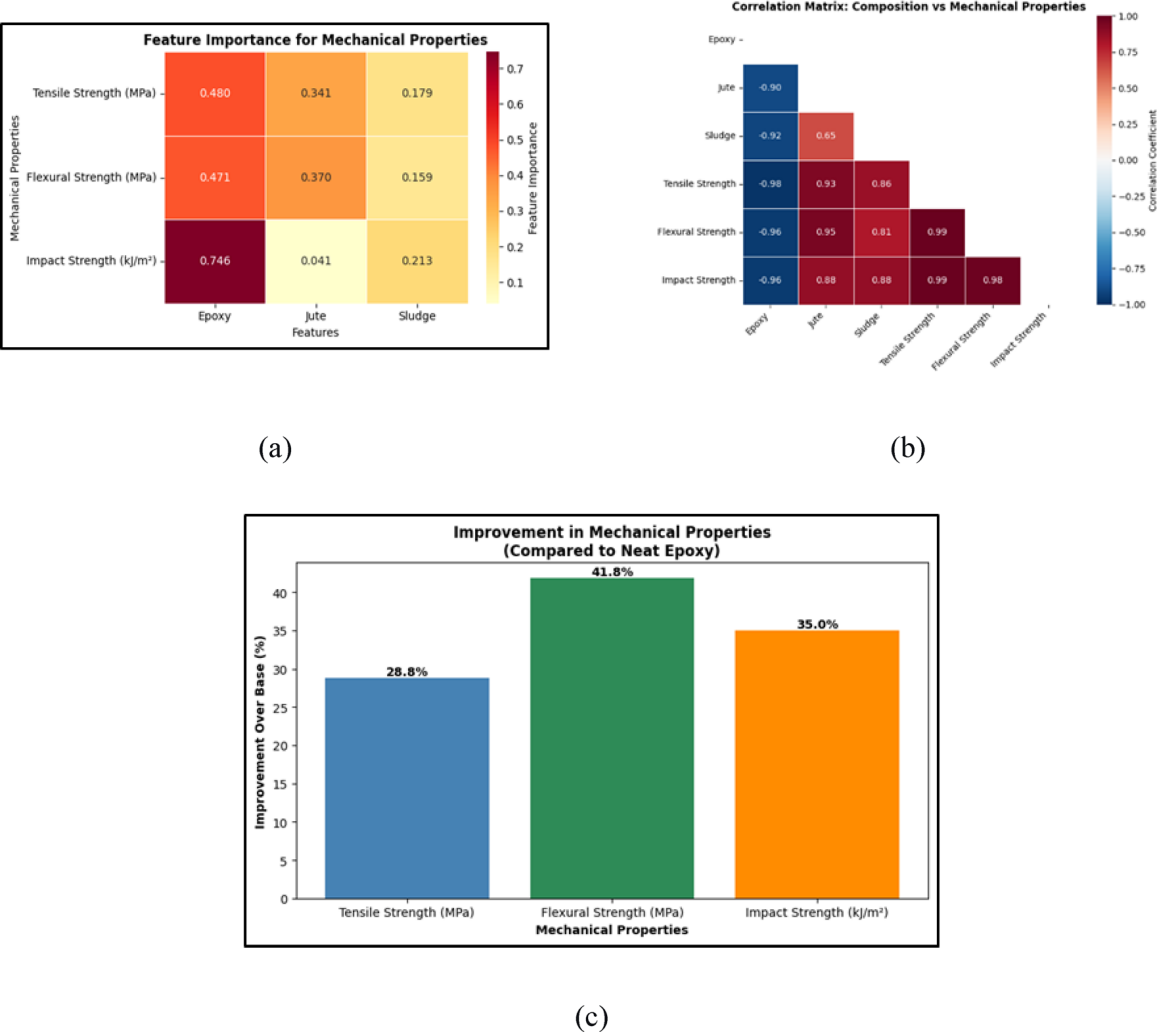
(**a**) Relative feature importance of input parameters influencing composite performance, (**b**) correlation matrix illustrating interdependence among processing variables and properties, and (**c**) optimization results identifying the optimal composite composition for enhanced performance.

The hierarchy of feature importance (epoxy > jute > sludge) as presented in Fig. 5a is an immediate depiction of the basic functions of each component of the composite. Epoxy prevails (importance: 0.48–0.75) since it is the continuous phase that carries the loads, maintains structural integrity, and facilitates energy dissipation (especially vital in impact resistance) as the matrix. The tensile (0.34) and flexural (0.37) strength are highly affected by jute fiber content owing to bridging and load-transfer mechanisms, but the impact strength (0.04) is not significantly affected since short natural fibers do not work well with dynamic loading. The lowest effect on strength (0.16–0.18) is exerted by LD sludge content since poor particle-matrix adhesion and stress concentration lead to reduced performance, although it contributes to improved impact strength (0.21) since hard particles can deflect cracks. The importance of this hierarchy is that mechanical properties are maximized at 20 wt% sludge: above this concentration, agglomeration of particles interferes with the predominant continuity of the epoxy matrix.

The significant contribution of jute fiber to tensile and flexural strength arises from efficient load transfer across the fiber–matrix interface. Fiber bridging and pull-out mechanisms enhance resistance to crack propagation, particularly under tensile and bending loads. Although sludge content has the smallest effect on strength properties (0.179 tensile and 0.159 flexural), it has a significant effect on impact strength (0.213), meaning that the hard sludge particles contribute to crack deflection and localized plastic deformation, though overloading the material above 20 wt% reduces interfacial adhesion and boosts brittleness as seen by the deterioration of mechanical properties at 25 wt% sludge.

### Correlation matrix

The correlation matrix shown in Fig. 5b indicates that negativities of composition are strong as the correlations between epoxy, jute, and sludge are high with negative correlation of − 0.92, − 0.90; this can be attributed to the fact that an increase in the percentage composition causes a reduction in the others as dictated by the formulation constraint: the total weight% has to be 100. This inherent trade-off is a direct effect on behavior of mechanical properties. All of the three mechanical properties, tensile strength (− 0.98), flexural strength (− 0.96), and impact strength (− 0.96) have very strong negative relationships with sludge content, which confirms that above the optimal sludge loading (20 wt%) the mechanical properties are reduced by lack of adhesion, and agglomeration of particles. On the other hand, tensile (0.98), flexural (0.96), and impact strength (0.96) exhibit very strong positive correlations with epoxy content, which highlights the importance of polymer matrix in the maintenance of structural integrity, transfer of stress, and toughness provided by good interfaces with both jute fibers and sludge particles. Another strong correlation can be found between jute fiber content and tensile (0.92), flexural strength (0.95) which indicates that jute fiber can reinforce the material through the mechanism of fiber-matrix interaction and load bearing, but the correlation between jute fiber content and impact strength is moderate (0.88), indicating that the role of jute fiber is not as dominant as that of the epoxy matrix under dynamic loading. These correlation trends indicate that there is an undeniable mechanistic trade-off that the more epoxy used, the better the mechanical strength achieved, but the more sludge used, though it is good in hardness and density, the more it negatively affects the strength properties beyond a certain optimal level, and this is the way formulation strategies can be pursued towards balanced performance in sustainable hybrid composites.

### Optimization of composite composition

The composite formula (S4) was optimized to reach high mechanical characteristics in comparison to the neat epoxy base, and the improved mechanical properties of the composite formula can be attributed to the synergistic reinforcement processes of the jute fiber and LD sludge (Fig. 5c). The flexural strength displayed maximum improvement of 41.8 which showed an increase of 22.44–31.81 MPa under bending due to high transfer of the load and bridging effect of the jute fibers which in turn reduced the concentration of stress and also delayed the formation of a crack, which is attributed to enhanced interfacial adhesion due to uniform dispersion of the sludge particles. It has increased tensile strength of 28.8% with the sludge increasing tensile strength of 48–61.84 MPa; this is mainly because of the reinforcing nature of jute fibers, which bear the axial loads, which in combination with both increase the continuity and resist tensile deformation of the matrix. Impact strength was enhanced by 35.0% to 18.026 kJ/m^2^ because of energy dissipation mechanisms (fiber pull-out, crack deflection around sludge particles, and matrix micro-cracking) leading to a significant increase in toughness. These percentage improvements represent the positive incorporation of natural fiber and industrial waste filler, with the jute being the main form of reinforcement, the sludge modifying microstructure and interfacial characteristics, and the epoxy matrix being the type of reinforcement that bonds the hybrid composite system, with the resulting tensile, flexural, and impact properties to be balanced.

Four models were considered in the study however, when it comes to the practical prediction task of similar small datasets, both Random Forest and XGBoost are the models to use because of their better generalization performance (as seen through cross-validation R^2^ = 0.93–0.94). Decision Tree and Gradient Boosting are included only to demonstrate how overfitting can occur when the models are memorized by the training data, leaving an important warning to researchers using ML on small experimental datasets.

## Conclusions

The current study was able to develop sustainable hybrid composites that were reinforced by jute fiber and LD sludge in an epoxy matrix, and it was evident that the mechanical performance was improved. The best composition (S4: 60 wt% epoxy, 20 wt% jute, 20 wt% LD sludge) was the one with maximum properties: tensile strength (48 MPa) increased to 61.84 MPa (28.8% higher), and flexural strength (22.44 MPa) increased to 31.81 MPa (41.8% higher). Such enhancements are explained by the successful fiber-matrix interaction, homogeneous dispersion of LD sludge, and efficient stress transfer mechanisms.

The addition of LD sludge to 25 wt% (S5) resulted in a decline in all properties due to particle agglomeration, weak interfacial bonding, and stress concentration, which indicates that 20 wt% LD sludge is the optimal filler loading.

In terms of machine learning, both Decision Tree and Gradient Boosting obtained a perfect fit to the training data (R^2^ = 1.0000 ), which is mostly due to memorization of the small sample (*n* = 6 formulations) and not the actual predictive ability. Random Forest showed better generalization with cross-validation R^2^ = 0.9395, and XGBoost also had a comparable cross-validation performance (R^2^ = 0.94 ± 0.04). These results should be taken with caution. Despite the good predictive strength of XGBoost on the training data, this is indicative of memorization due to the small size of the dataset. Thus, the claims about the inverse design capability are premature.

The model can be considered as a demonstration-of-concept that machine learning can learn composition-property relationships in a small experimental space. Two important conditions must be satisfied before it can be practically used as an inverse design tool: (1) training on a significantly large dataset (at least 30–50 formulations) to learn generalizable patterns; and (2) independent experimental validation of unseen compositions that the training range does not cover. Future directions must focus on producing bigger datasets, and external validation before asserting predictive design ability.

This study demonstrates that industrial waste-filled EP-JF composites can be utilized as low-cost materials, contributing to sustainable material development. The optimised composite system exhibits enhanced mechanical properties, making it a suitable candidate for load-bearing and semi-structural applications such as interior automotive panels, lightweight housings, brackets, enclosures, partition boards, furniture components, building panels, and other structural components where moderate strength, stiffness, and durability are required.

### Limitations and future scope of this work

This research has weaknesses in the limited compositional range explored (six formulations of fixed jute content) and the rather small dataset on which machine learning models are trained, which limits more extensive generalization. Also, the evaluation of the machine learning models was done based on training only and no independent test set was used; hence, the performance measures reported indicate model performance on the experimental data and not predictive ability on unknown compositions. Besides, the machine learning model was trained on a controlled experimental dataset in which fiber length, fiber surface treatment and processing conditions were kept at constant values. As a result, the model can only predict composites prepared under these particular conditions and the overall applicability of the model to composites with different fiber properties or processing requirements is not determined. The work was mainly on mechanical properties, but no evaluation of long-term durability, thermal behavior, and large-scale manufacturing feasibility was done in details. More research with broader compositional space, larger data, test validation, longer performance testing, and inclusion of other input parameters like fiber length, surface treatment, and processing conditions is necessary to enhance practical utility and more physically interpretable and broadly generalizable models.

The exclusion of processing parameters (e.g., fiber length, interfacial bonding, and porosity) may limit the physical interpretability of the ML model. Future studies will incorporate these variables to develop more robust and physically informed predictive models.

## Data Availability

The data used and/or analyzed during the current study are available from the corresponding author upon reasonable request.
